# Supplementary material for: Energy-saving hydrogen production by chlorine-free hybrid seawater splitting coupling hydrazine degradation
Source: Nat Commun. 2021 Jul 7;12:4182. doi: 10.1038/s41467-021-24529-3 (PMC8263752; doi:10.1038/s41467-021-24529-3)
Supplement: Supplementary file 1 — Supporting Information [file 41467_2021_24529_MOESM1_ESM.pdf]

# Supplementary Information

# Energy-saving Hydrogen Production by Chlorine-free Hybrid Seawater Splitting Coupling Hydrazine Degradation

Fu Sun,[1] Jingshan Qin,[2] Zhiyu Wang,[1,*] Mengzhou Yu,[3] Xianhong Wu,[1] Xiaoming Sun[2,*] and Jieshan Qiu[1,4,*]

[1] State Key Lab of Fine Chemicals, Liaoning Key Lab for Energy Materials and Chemical Engineering, PSU-DUT Joint Center for Energy Research, Dalian University of Technology, Dalian 116024, China

[2] College of Chemistry, Beijing University of Chemical Technology, Beijing 100029, China

[3] State Key Laboratory of Space Power-Sources Technology, Shanghai Institute of Space Power-Sources, Shanghai 200245, China

[4] College of Chemical Engineering, Beijing University of Chemical Technology, Beijing 100029, China

* E-mail: zywang@dlut.edu.cn, sunxm@mail.buct.edu.cn, jqiu@dlut.edu.cn

## Methods

**Calculation of ECSA.** The ECSA of the electrode was calculated by the equation:

$$\mathrm{ECSA} = RF \times A_{\mathrm{geo}} \quad (1)$$

where $A_{\mathrm{geo}}$ is the geometric surface area of the electrode and $RF$ is the roughness factor. The $RF$ was estimated by electrical double layer capacitance ($C_{\mathrm{dl}}$) of the electrode by the equation:

$$RF = C_{\mathrm{dl}} / C_{\mathrm{s}} \quad (2)$$

The $C_{dl}$ was measured by CVs in a non-Faradaic region at various scan rates. The specific capacitance ($C_s$) refers to the capacitance of the materials with a smooth planar surface under identical electrolyte conditions and a typical value is 0.04 mF $cm^{-2}$ [1,2].

**TOF calculation.** The TOF of HER was calculated according to the equation:

$$\mathrm{TOF} = j / (2 \times n \times F) \quad (3)$$

where $j$ (A $cm^{-2}$) is the current density, $F$ is the Faraday constant (96485 C $mol^{-1}$), $n$ is the number of active sites (mol $cm^{-2}$). The $n$ was obtained by the CVs in a potential range of 0 – 0.6 V (*vs.* RHE) in 1.0 M PBS at a scan rate of 50 mV $s^{-1}$ [3,4]. The surface charge density ($Q_s$, C $cm^{-2}$) was calculated as half of the integrated charge of CVs. The $n$ can be calculated by the equation:

$$n = Q_s / F \quad (4)$$

**Detection of hypochlorite.** The hypochlorite concentration in the electrolyte during long-term seawater electrolysis was measured by *o*-tolidine method via the redox reaction between hypochlorite and *o*-tolidine[5]. The testing solution was 0.5 mL *o*-tolidine in DI water (10 mL). To obtain the calibration curve, different amounts of $Cl_2$ (2.5, 5, 10, 15, and 25 μL) freshly generated by reacting $KMnO_4$ (500 mg) with HCl (6.0 M, 10 mL) were collected into KOH solution (1.0 M, 0.1 mL) and injected into the testing solution. The UV-vis spectrum was measured to obtain the $ClO^-$ concentration-absorbance curve at $\lambda$ = 436 nm. After long-term seawater electrolysis, a part of the electrolyte (0.1 mL) was added into the testing solution, in which the $ClO^-$ concentration can be determined by comparing the UV-vis spectrum and calibration curve.

**Calibration of the reference electrode.** The reference electrode was calibrated in high purity $H_2$ saturated electrolyte with Pt as the working electrode. The cyclic voltammograms (CVs) were measured at a scan rate of 1.0 mV $s^{-1}$, and the average of the two potentials at which the current crossed zero was taken as the thermodynamic potential (*vs.* Ag/AgCl electrode) for the hydrogen electrode reactions.

**Cost analysis of hybrid seawater electrolysis.** Use of commercial hydrazine leads to a hydrogen cost of *ca.* 2.38 USD per $m^3$ $H_2$ assuming an average electricity cost of 0.11 USD per kWh in China and hydrazine (80 %) cost of 2,415 USD per ton (price at Apr 2020). Electrolysis of costless hydrazine sewage can largely reduce this cost to below

0.37 – 0.42 USD. It far excels existing water and seawater electrolyzers suffering the additional cost of seawater desalination and frequent anode maintenance besides unaffordable electricity consumption. The expense of our technique is anticipated to be even reduced by coupling renewable solar or wind power into the system. Meanwhile, the extra function of our hybrid electrolyzer in toxic hydrazine treatment further adds inestimable benefits to the protection of the ecosystem and human health. This merit is hardly realized by conventional ones with less environmental sustainability.

**Computational methods.** Spin-polarized first-principles calculations were performed in terms of density functional theory (DFT) implemented in the Vienna ab initio simulation package (VASP)[6]. We used the plane-wave basis set with an energy cutoff of 400 eV, projector augmented wave (PAW) pseudopotentials and the generalized gradient approximation parameterized by Perdew, Burke, and Ernzerhof (GGA-PBE) for exchange-correlation functional[7,8]. The equilibrium lattice constant of *fcc* unit cell of NiCo alloy with a Ni : Co ratio of 3 : 1 was optimized to be 3.478 Å by using a 15 × 15 × 15 Monkhorst-Pack *k*-point grid for Brillouin zone sampling. It was then used to construct the model of the (111), (110) and (100) surface of $Ni_3Co$ alloy. During structural optimizations, the gamma point in the Brillouin zone was used for *k*-point sampling, and the bottom stoichiometric layer was fixed while the top two were allowed to fully relax. The model structures were fully optimized for the ionic and electronic degrees of freedom using the convergence criteria of $10^{-5}$ eV for electronic energy and 0.02 eV/Å for the forces on each atom. Grimme's semiempirical DFT-D3 scheme of dispersion correction was adopted to describe the van der Waals (vdW) interactions in layered materials[9].

The oxidation of hydrazine into nitrogen occurs in the following six consecutive elementary steps:

$$\text{(A)}\ * + N_2H_4 \rightarrow N_2H_4* \quad (5)$$

$$\text{(B)}\ N_2H_4* \rightarrow N_2H_3* + H^+ + e^- \quad (6)$$

$$\text{(C)}\ N_2H_3* \rightarrow N_2H_2* + H^+ + e^- \quad (7)$$

$$\text{(D)}\ N_2H_2* \rightarrow N_2H* + H^+ + e^- \quad (8)$$

$$\text{(E)}\ N_2H* \rightarrow N_2* + H^+ + e^- \quad (9)$$

$$\text{(F)}\ N_2* \rightarrow * + N_2 \quad (10)$$

The asterisk (*) represents the reaction surface of calculated $Ni_3Co$ alloy (111), (110) and (100) facets. The $N_2H_4$*, $N_2H_3$*, $N_2H_2$*, $N_2H$* and $N_2$* denote the models with the corresponding chemisorbed species on reaction surfaces. The Gibbs free energy change ($\Delta G$) is defined as:

$$\Delta G = \Delta E + \Delta \mathrm{ZPE} - T\Delta S + \Delta G_{\mathrm{U}} + \Delta G_{\mathrm{pH}} \tag{11}$$

where $\Delta E$, $\Delta$ZPE, $\Delta S$ are the differences of DFT total energy, zero-point energy, entropy, respectively. The $T$ is the temperature. The $\Delta G_{\mathrm{U}}$ and $\Delta G_{\mathrm{pH}}$ are the free energy contributed by the electrode potential and $H^+$ concentration, respectively. They were set to zero[10,11]. The values of $\Delta$ZPE and $\Delta S$ for the gas phase $H_2$, $N_2$ and $N_2H_4$ were obtained from the NIST-JANAF thermodynamics table and the contribution of vibration of all adsorbed species was considered in our calculation[12]. Using the computational hydrogen electrode (pH = 0, $p$ = 1 atm, $T$ = 298 K), the Gibbs free energy of $H^+ + e^-$ was replaced implicitly with the Gibbs free energy of a 1/2 $H_2$ molecule[13]. Thus $\Delta G$ of the stepwise reaction can be calculated by the equations:[11,14]

$$\Delta G_{\mathrm{A}} = \Delta G_{*\mathrm{N_2H_4}} - \Delta G_{*} - \Delta G_{\mathrm{N_2H_4}} \tag{12}$$

$$\Delta G_{\mathrm{B}} = \Delta G_{*\mathrm{N_2H_3}} + 0.5\ \Delta G_{\mathrm{H_2}} - \Delta G_{*\mathrm{N_2H_4}} \tag{13}$$

$$\Delta G_{\mathrm{C}} = \Delta G_{*\mathrm{N_2H_2}} + 0.5\ \Delta G_{\mathrm{H_2}} - \Delta G_{*\mathrm{N_2H_3}} \tag{14}$$

$$\Delta G_{\mathrm{D}} = \Delta G_{*\mathrm{N_2H}} + 0.5\ \Delta G_{\mathrm{H_2}} - \Delta G_{*\mathrm{N_2H_2}} \tag{15}$$

$$\Delta G_{\mathrm{E}} = \Delta G_{*\mathrm{N_2}} + 0.5\ \Delta G_{\mathrm{H_2}} - \Delta G_{*\mathrm{N_2H}} \tag{16}$$

$$\Delta G_{\mathrm{F}} = \Delta G_{*} + \Delta G_{\mathrm{N_2}} - \Delta G_{*\mathrm{N_2}} \tag{17}$$

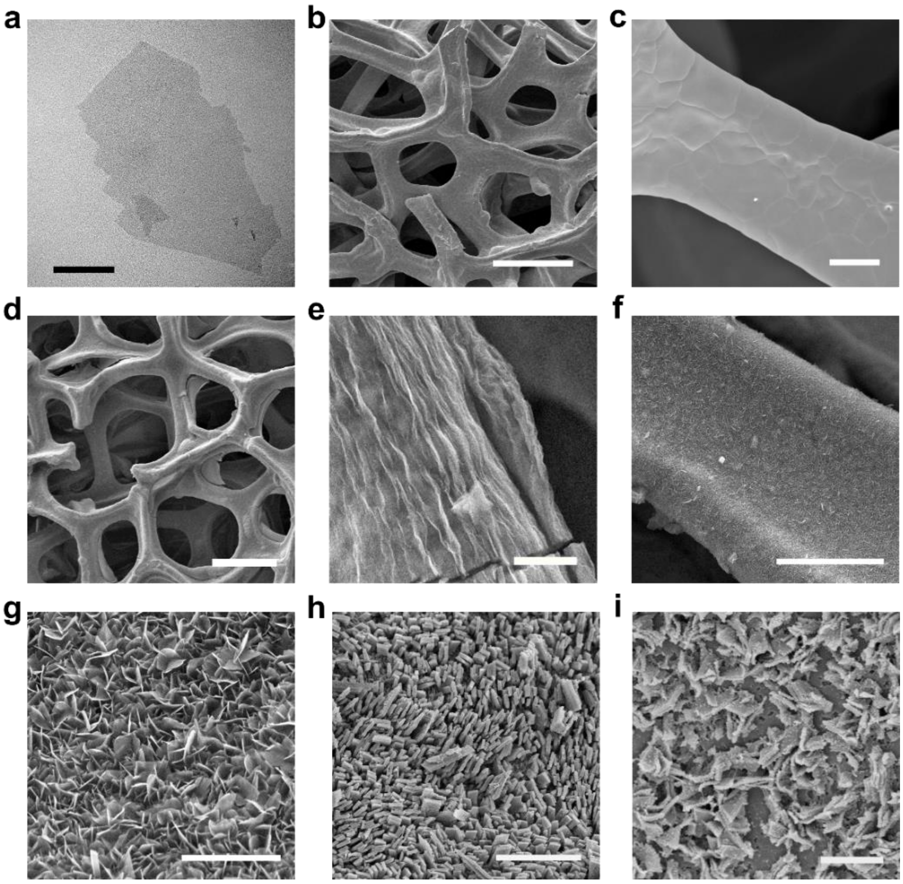


**Supplementary Figure 1.** (a) TEM image of pristine $Ti_3C_2T_x$ MXene. SEM images of (b, c) bare CF, (d, e) MXene/CF, (f, g) NiCo-MOF/MXene/CF, (h) NiCo-MOF/CF and (i) NiCo@C/CF. Scale bar, (a) 100 nm; (b, d) 100 µm; (c) 20 µm; (e) 10 µm;(f) 30 µm; (g, h) 5 µm; (i) 2 µm.

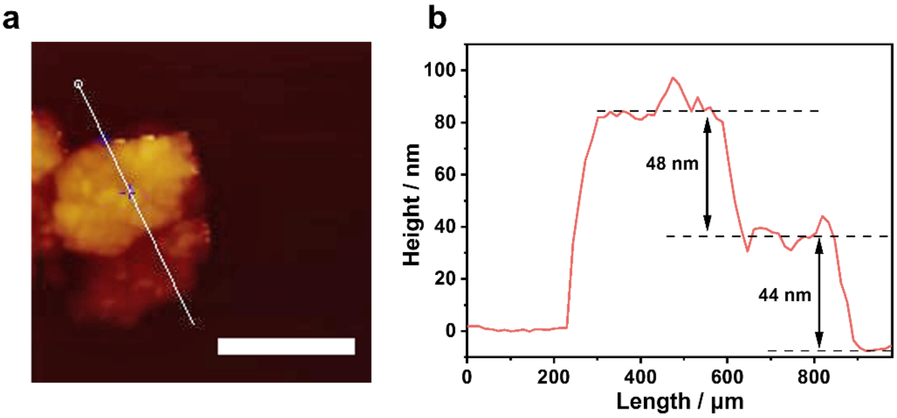


**Supplementary Figure 2.** (a) AFM image of NiCo@C nanosheets peeled off from the NiCo@C/MXene/CF. Scale bar: 500 nm. (b) Thickness curves of NiCo@C nanosheets.

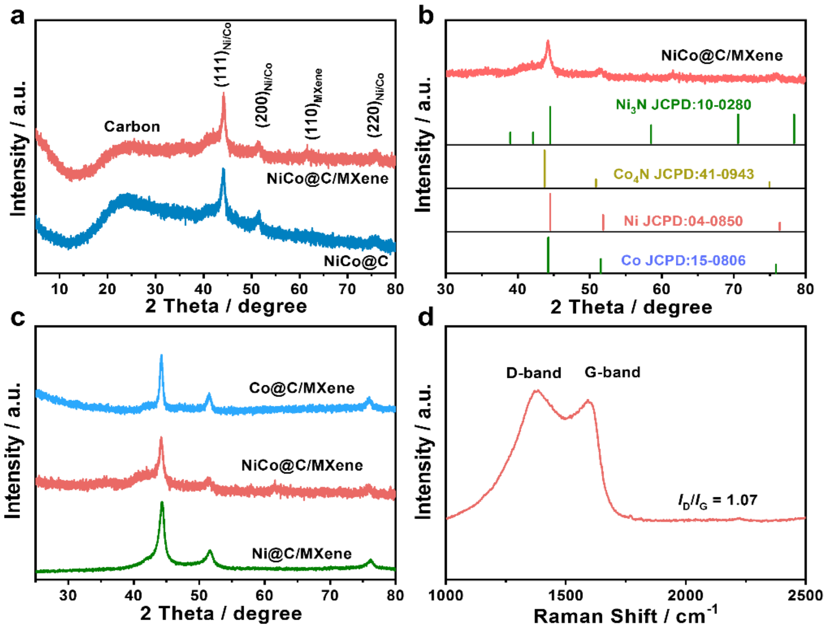


**Supplementary Figure 3.** XRD patterns of (a) NiCo@C and NiCo@C/MXene peeled off from the CF. (b) XRD patterns of NiCo@C/MXene peeled off from the CF. The standard patterns of $Ni_3N$, $Co_4N$, Ni and Co are also presented for comparison. (c) XRD patterns of Ni@C/MXene, Co@C/MXene and NiCo@C/MXene. (d) Raman spectrum of NiCo@C/MXene/CF.

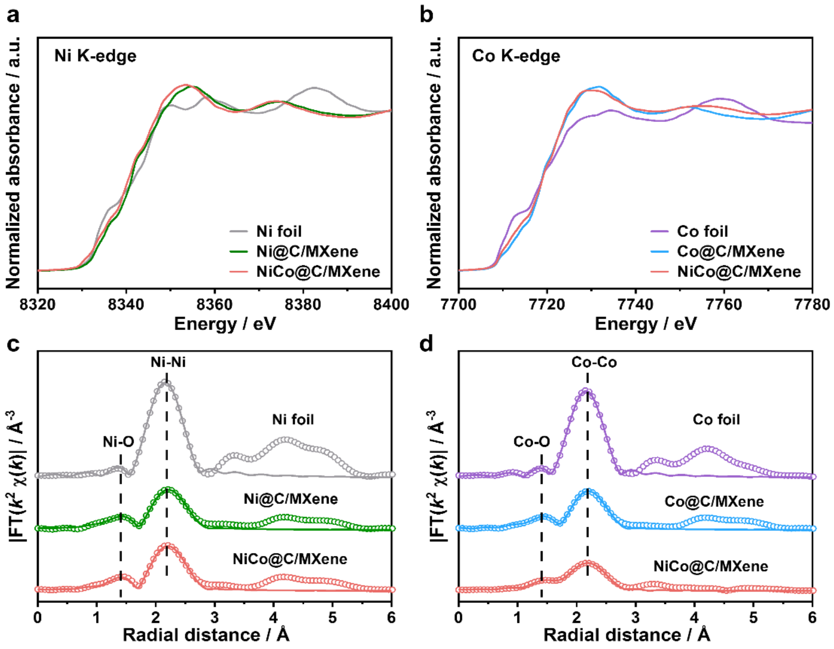


**Supplementary Figure 4.** (a, c) The normalized Ni K-edge XANES and the corresponding $k^2$-weighted FT-EXAFS spectra of NiCo@C, Ni@C and Ni foil. (b, d) The normalized Co K-edge XANES and the corresponding $k^2$-weighted FT-EXAFS spectra of NiCo@C, Co@C and Co foil.

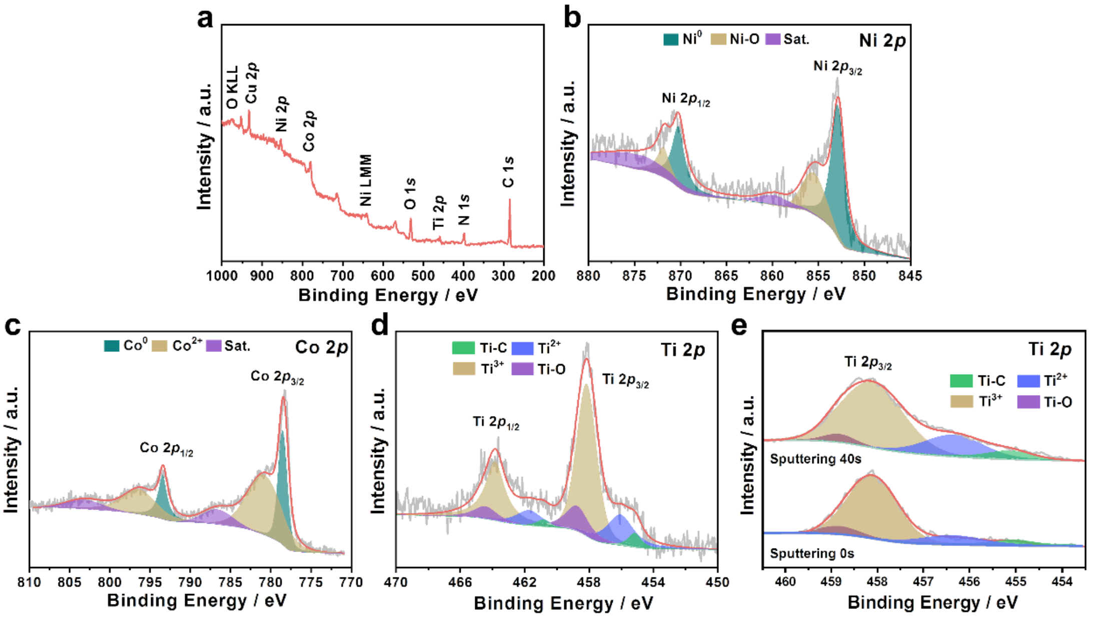


**Supplementary Figure 5.** (a) XPS survey scan, (b) Ni 2*p*, (c) Co 2*p* and (d) Ti 2*p* spectrum of NiCo@C/MXene/CF. (e) Ti 2*p* XPS spectra of NiCo@C/MXene/CF after $Ar^+$ sputtering.

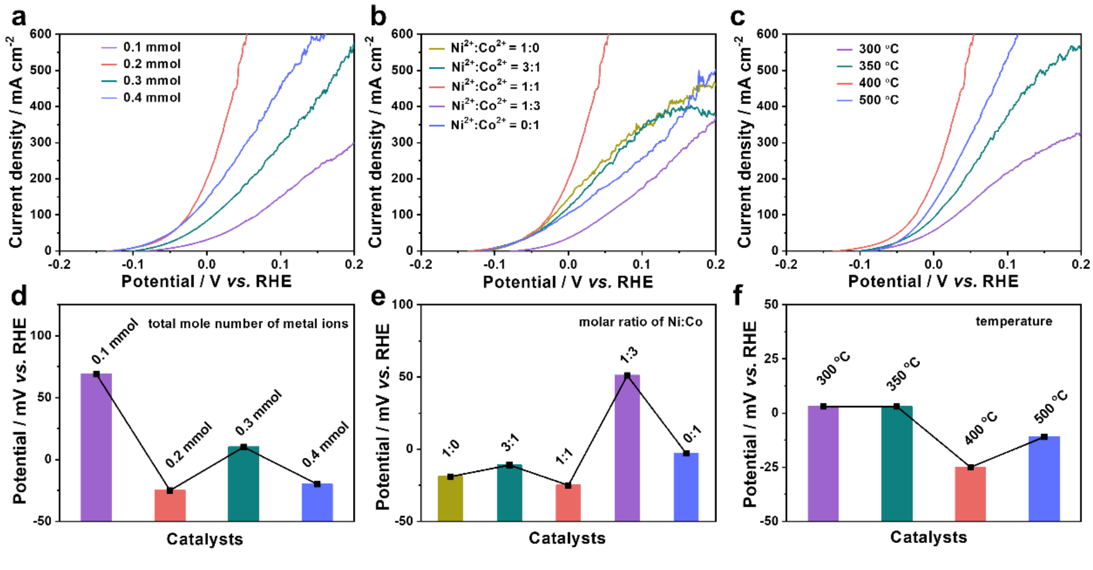


**Supplementary Figure 6.** The LSV curves of NiCo@C/MXene/CF obtained at different (a) metal ion concentrations, (b) molar ratios of $Ni^{2+}$ and $Co^{2+}$ in precursor and (c) annealing temperatures. (d–f) A comparison of these catalysts in the potential required to achieve a current density of 100 mA $cm^{-2}$ for HzOR. All the tests are conducted at a scan rate of 10 mV $s^{-1}$ in 1.0 M KOH with 0.5 M $N_2H_4$.

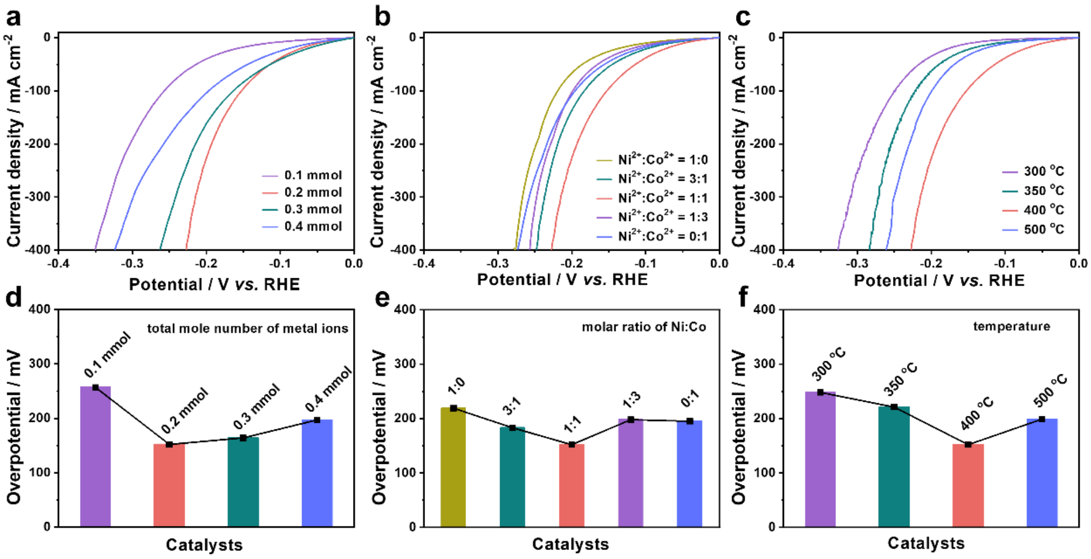


**Supplementary Figure 7.** The LSV curves of NiCo@C/MXene/CF obtained at different (a) metal ion concentrations, (b) molar ratio of $Ni^{2+}$ and $Co^{2+}$ in precursor and (c) annealing temperatures. (d–f) A comparison of these catalysts in the potential required to achieve a current density of 100 mA cm$^{-2}$ for HER. All the tests are conducted at a scan rate of 10 mV s$^{-1}$ in 1.0 M KOH.

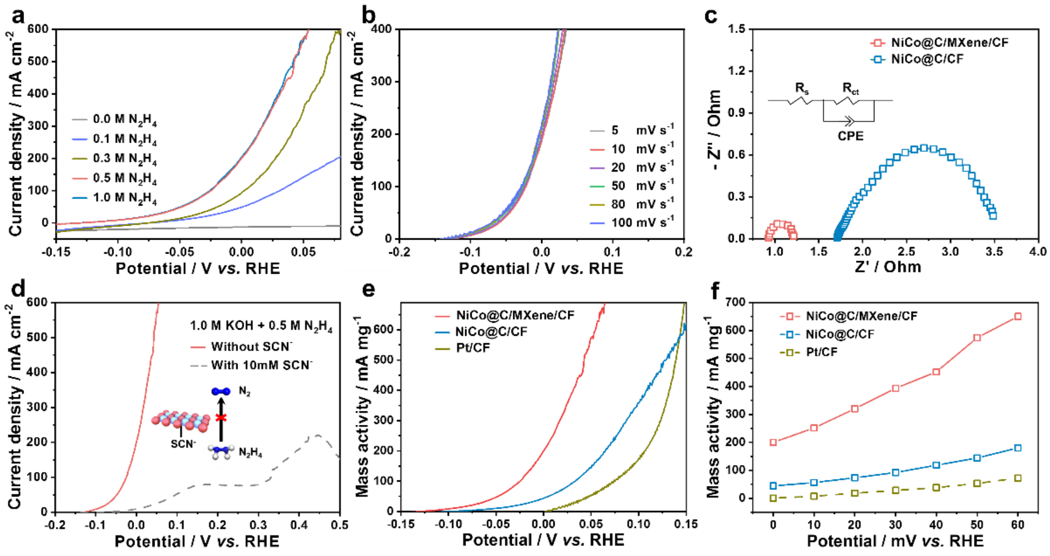


**Supplementary Figure 8.** (a) LSV curves of NiCo@C/MXene/CF for HzOR in 1.0 M KOH with various $N_2H_4$ concentrations. (b) LSV curves of NiCo@C/MXene/CF for HzOR at different scan rates. (c) EIS patterns of NiCo@C/MXene/CF and NiCo@C/CF for HzOR at 0.123 V *vs.* RHE. (d) LSV curves of NiCo@C/MXene/CF for HzOR with and without adding 10 mM SCN$^-$. (e, f) Mass activity of NiCo@C/MXene/CF, NiCo@C/CF and Pt/CF for HzOR. All the tests are conducted in 1.0 M KOH with 0.5 M $N_2H_4$ unless otherwise specified.

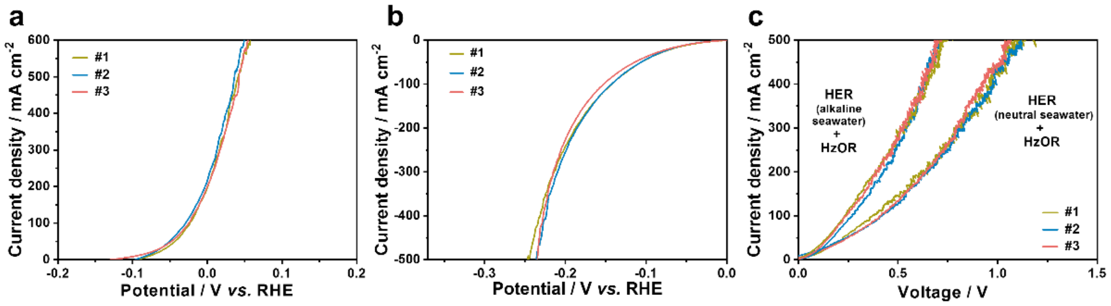


**Supplementary Figure 9.** The reproducibility LSVs of NiCo@C/MXene/CF electrode for (a) HzOR, (b) HER and (c) HER coupled HzOR for three tests.

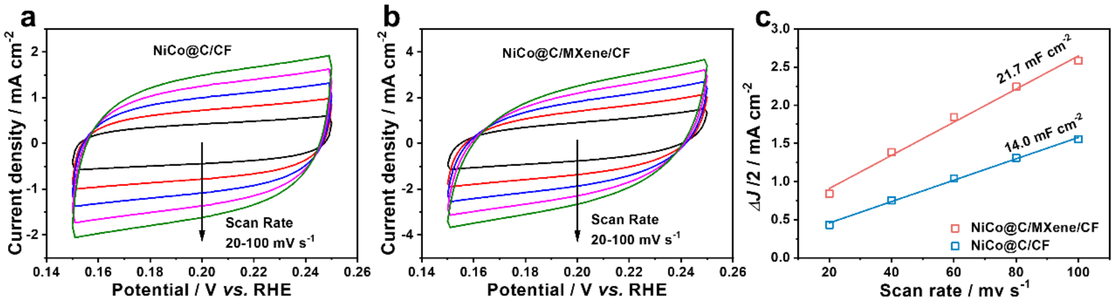


**Supplementary Figure 10.** The CVs of (a) NiCo@C/CF and (b) NiCo@C/MXene/CF in 1.0 M KOH. (c) The $C_{dl}$ of NiCo@C/MXene/CF and NiCo@C/CF determined by the plots of $\Delta j/2$ against various scan rates at 0.2 V vs. RHE. The $\Delta j$ is the difference between anodic and cathodic current densities in CVs at different scan rates at this potential.

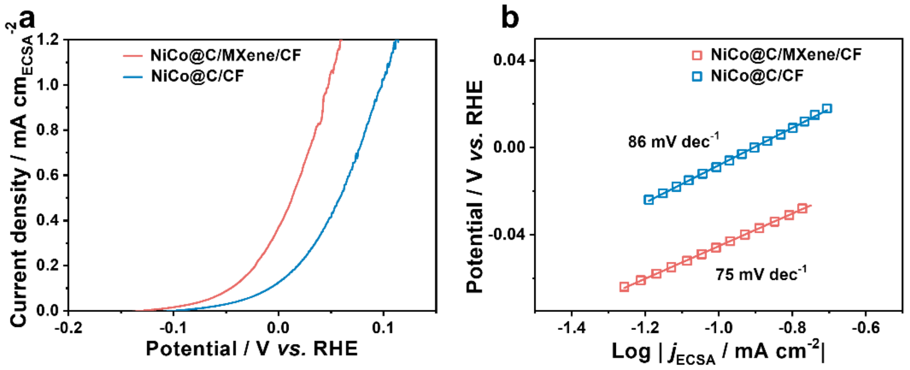


**Supplementary Figure 11.** (a) ECSA-normalized LSV curves of NiCo@C/MXene/CF and NiCo@C/CF for HzOR in 1.0 M KOH with 0.5 M $N_2H_4$. (b) Tafel plots of both electrodes derived from ECSA-normalized LSV curves.

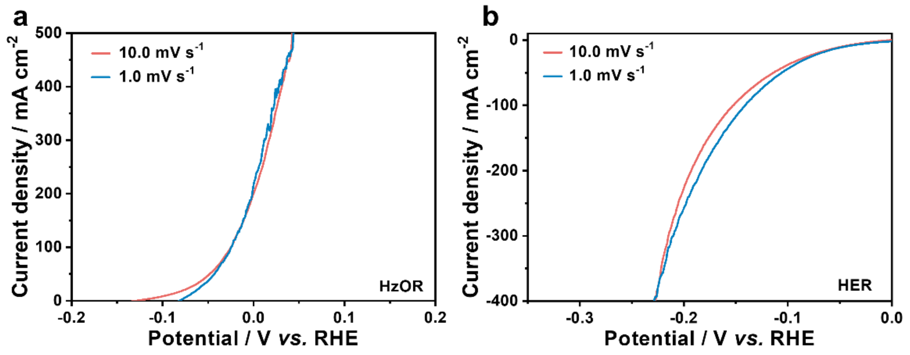


**Supplementary Figure 12.** A comparison of the LSVs of NiCo@C/MXene/CF for (a) HzOR in 1.0 M KOH with 0.5 M $N_2H_4$ and (b) HER in 1.0 M KOH at the scan rate of 1.0 and 10.0 mV $s^{-1}$.

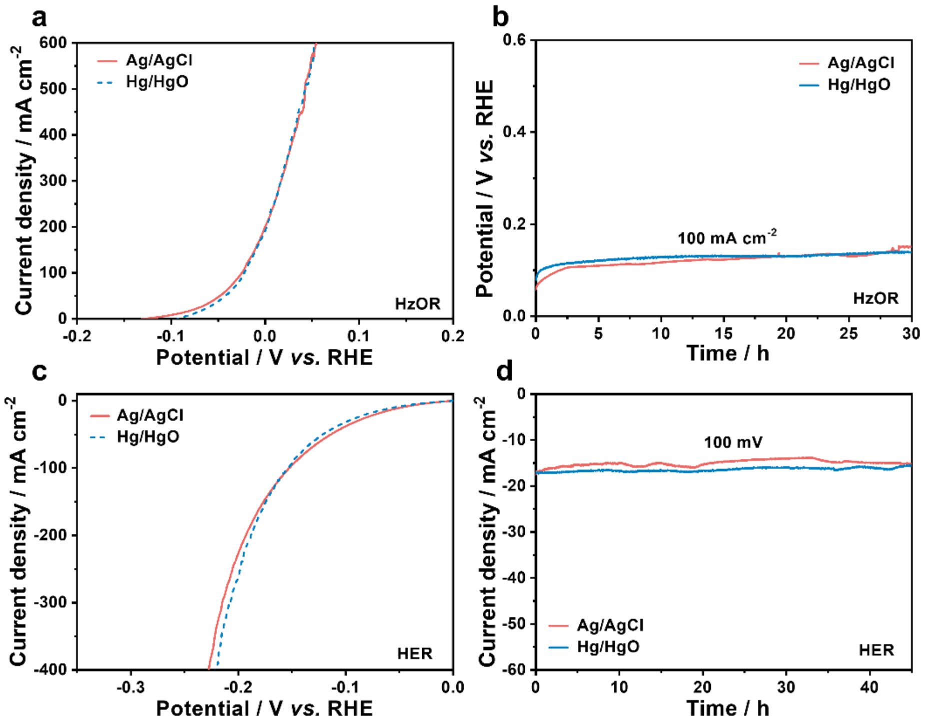


**Supplementary Figure 13.** A comparison of the catalytic performance of NiCo@C/MXene/CF, which are measured by using Ag/AgCl or Hg/HgO as the reference electrode. (a) LSVs and (b) chronopotentiometric curves at a current density of 100 mA $cm^{-2}$ for HzOR; (c) LSVs and (d) chronoamperometric curves for HER at $\eta$ = 100 mV.

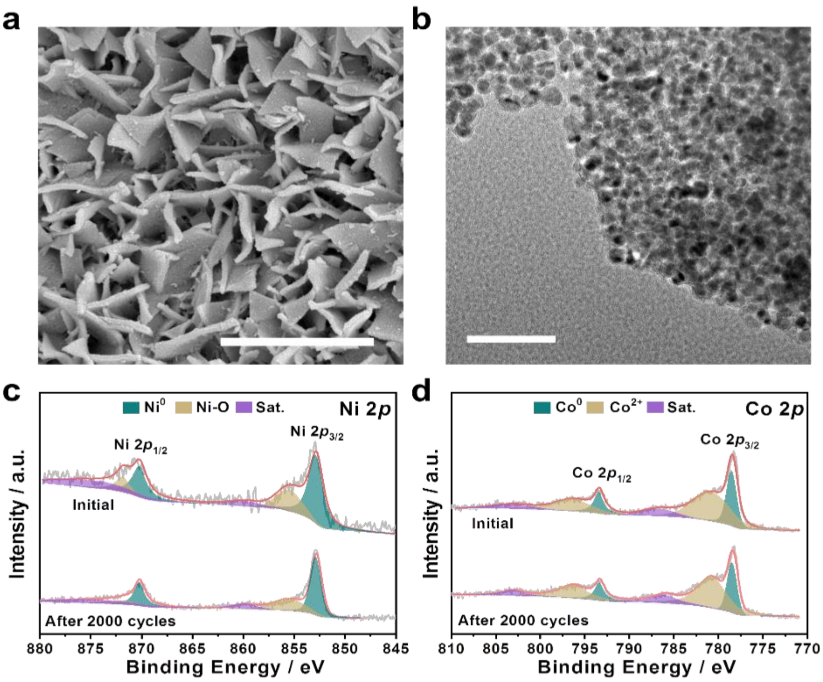


**Supplementary Figure 14.** (a) SEM and (b) TEM image of NiCo@C/MXene/CF after accelerated durability test for HzOR in 1.0 M KOH with 0.5 M $N_2H_4$. (c) Ni 2*p* and (d) Co 2*p* XPS spectra of NiCo@C/MXene/CF before and after HzOR. Scale bar, (a) 2 μm; (b) 50 nm.

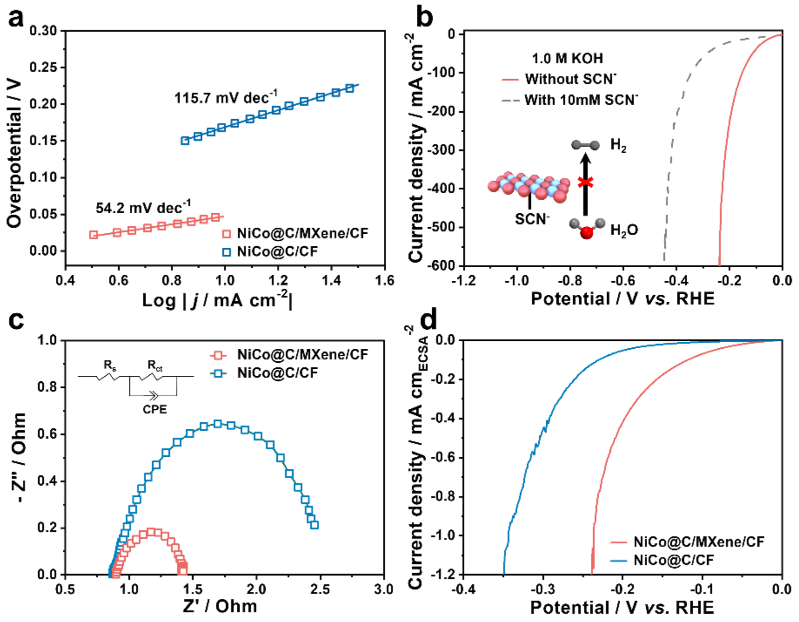


**Supplementary Figure 15.** (a) Tafel plots of NiCo@C/MXene/CF and NiCo@C/CF for HER. (b) LSV curves of NiCo@C/MXene/CF for HER with and without adding 10 mM $SCN^-$. (c) EIS patterns of NiCo@C/MXene/CF and NiCo@C/CF for HER at $\eta$ = 200 mV. (d) ECSA-normalized LSV curves of NiCo@C/MXene/CF and NiCo@C/CF for HER. All the tests are conducted in 1.0 M KOH.

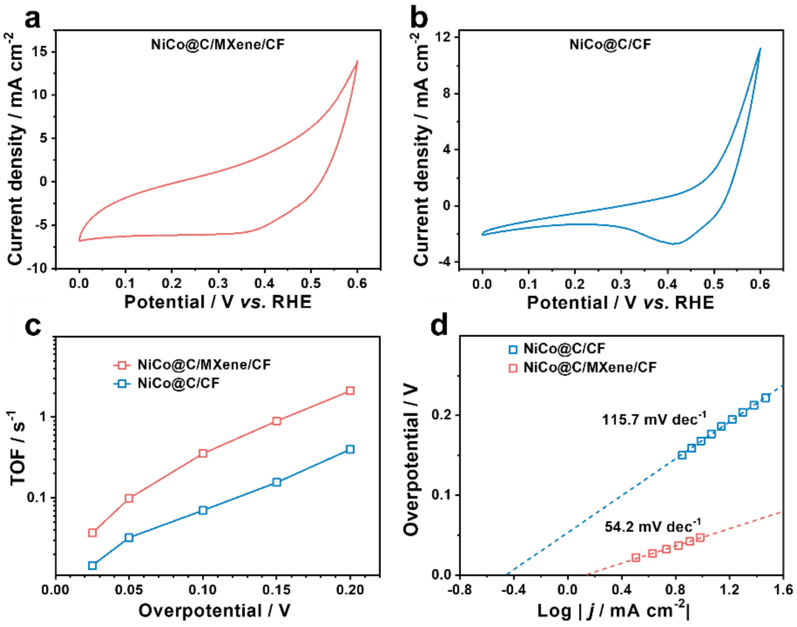


**Supplementary Figure 16.** The CVs of (a) NiCo@C/MXene/CF and (b) NiCo@C/CF in 1.0 M PBS at a scan rate of 50 mV $s^{-1}$. (c) TOF values of NiCo@C/MXene/CF and NiCo@C/CF for HER at various overpotentials in 1.0 M KOH. (d) Tafel plots of both electrodes in the region of low current densities for HER in 1.0 M KOH**.** The exchange current density is derived from the intercept of their extended line on the *x*-axis.

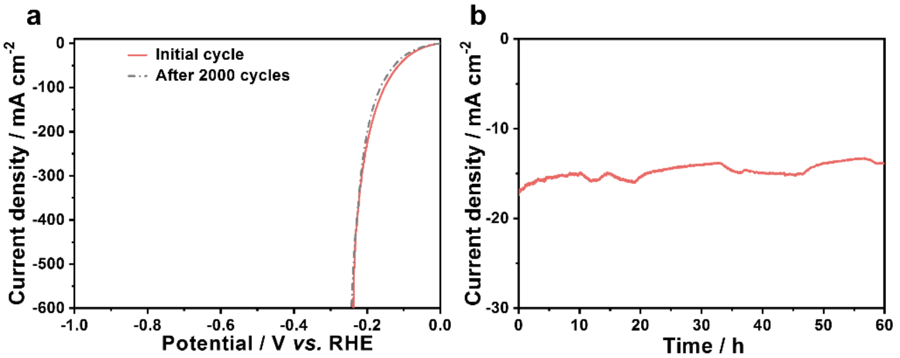


**Supplementary Figure 17.** (a) LSV curves of NiCo@C/MXene/CF for HER initially and after 2,000 sweeps at a scan rate of 50 mV $s^{-1}$. (b) Chronoamperometric curves of NiCo@C/MXene/CF for HER at $\eta = 100$ mV. All the tests are conducted in 1.0 M KOH.

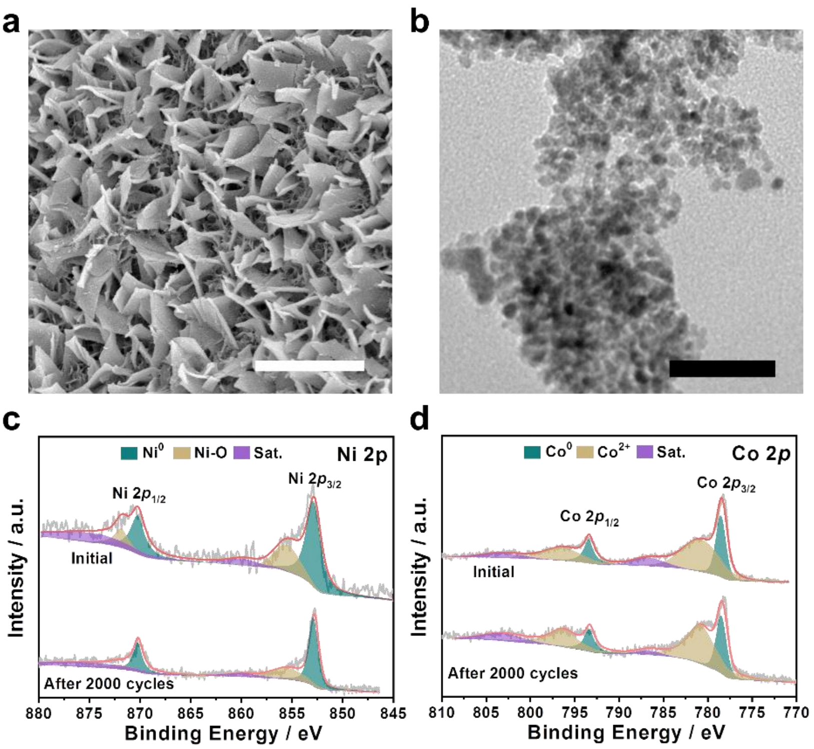


**Supplementary Figure 18.** (a) SEM and (b) TEM image of NiCo@C/MXene/CF after accelerated durability test for HER in 1.0 M KOH. (c) Ni 2*p* and (d) Co 2*p* XPS spectra of NiCo@C/MXene/CF before and after HER. Scale bar, (a) 2 μm; (b) 50 nm.

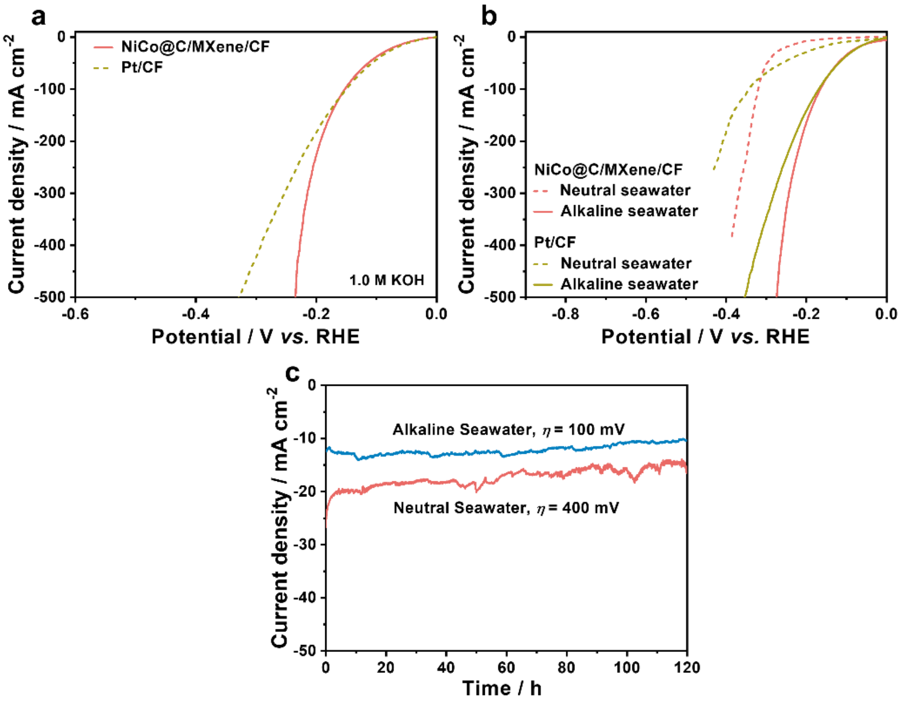


**Supplementary Figure 19.** LSV curves of NiCo@C/MXene/CF and 20 wt.% Pt loaded on CF (Pt/CF) for HER in (a) 1.0 M KOH and (b) neutral or alkaline seawater with 1.0 M KOH. (c) Chronoamperometric curves of NiCo@C/MXene/CF for HER in neutral seawater at $\eta$ = 400 mV and alkaline seawater with 1.0 M KOH at $\eta$ = 100 mV.

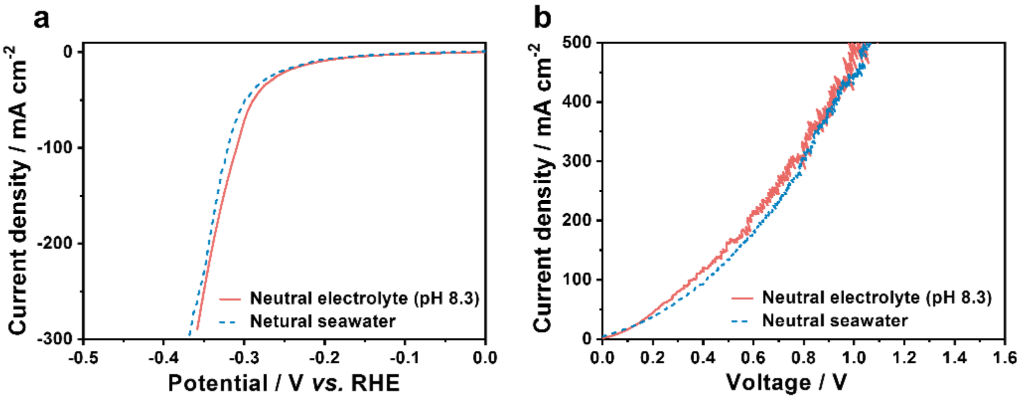


**Supplementary Figure 20.** (a) The LSVs of NiCo@C/MXene/CF for HER in neutral seawater and the seawater pH (8.3) mimicked electrolyte. (b) The LSVs of the hybrid electrolyzer using seawater pH (8.3) mimicked catholyte.

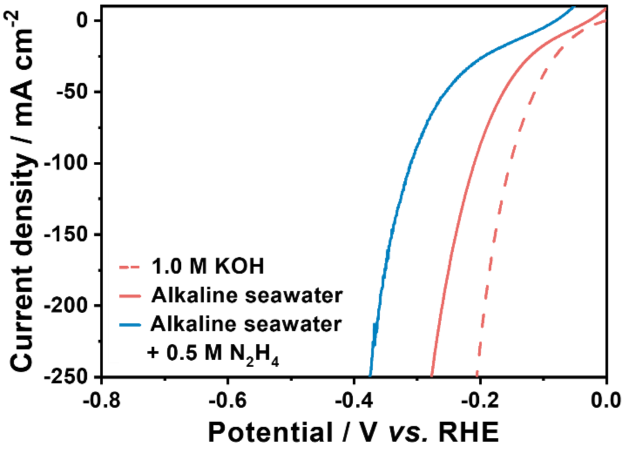


**Supplementary Figure 21.** The LSV curves of NiCo@C/MXene/CF for HER in 1.0 M KOH or alkaline seawater with and without 0.5 M $N_2H_4$.

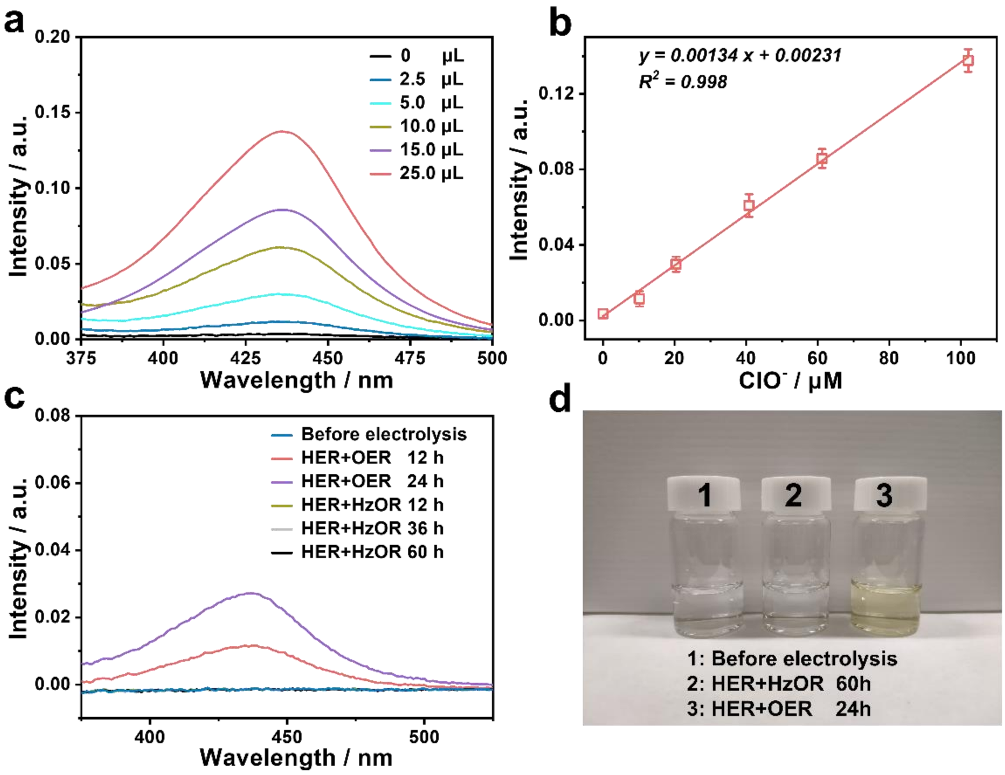


**Supplementary Figure 22.** (a) UV-vis absorption spectra of the testing solution containing 0.5 mL *o*-tolidine in DI water (10 mL) with different hypochlorite concentrations, which are obtained by collecting different volumes of $Cl_2$ (2.5, 5, 10, 15, and 25 μL) into 1.0 M KOH followed by injecting it into the testing solution. (b) The calibration curve is obtained by plotting the hypochlorite concentration against absorption peak intensity at $\lambda = 436$ nm. (c) UV-vis absorption spectra and (d) optical image of the testing solution after adding the electrolyte (0.1 mL) taken from various electrolysis systems after certain times into the testing solution.

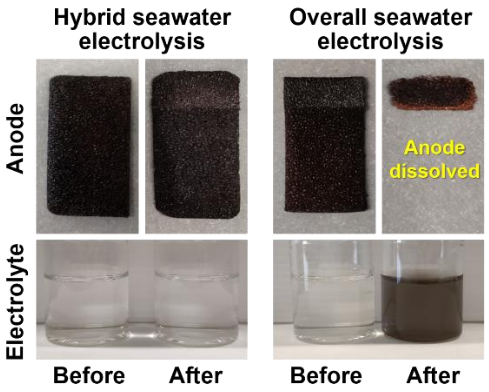


**Supplementary Figure 23.** Optical images of the anode and anolyte before and after seawater electrolysis in HSE or ASE for 60 h at 100 mA $cm^{-2}$.

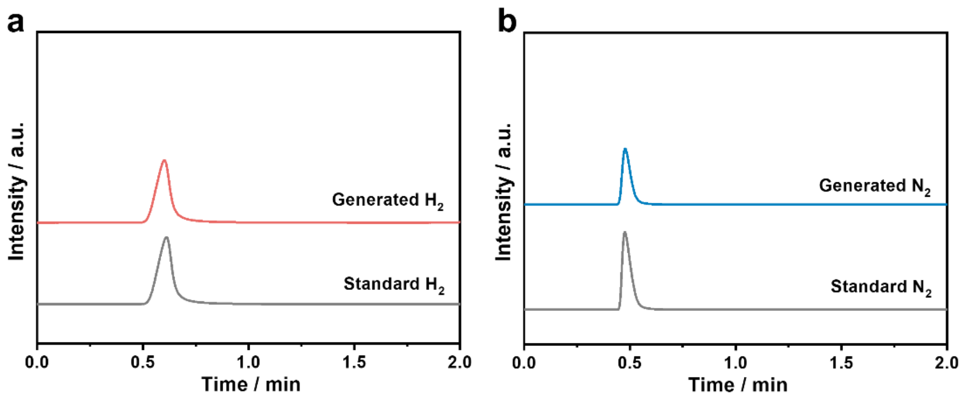


**Supplementary Figure 24.** The GC signals of gas products released from the (a) cathode and (b) anode chamber of hybrid seawater electrolyzer.

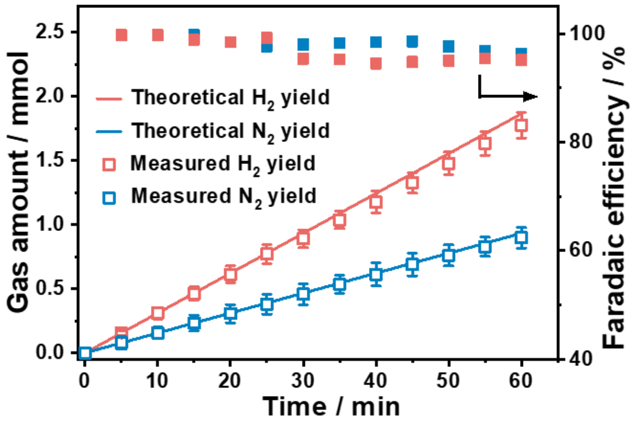


**Supplementary Figure 25.** The Faradaic efficiency of HzOR and HER in HSE at a fixed current of 100 mA.

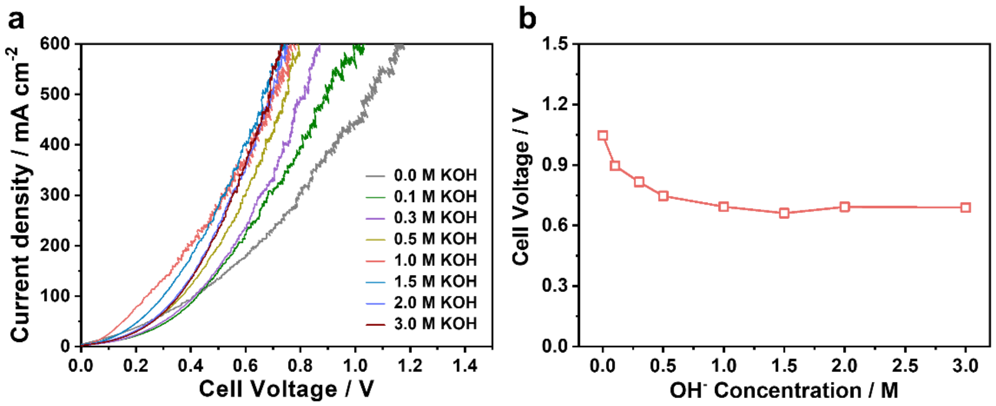


**Supplementary Figure 26.** (a) The LSVs of hybrid seawater electrolyzer using seawater with various $OH^-$ concentrations as the catholyte and 1.0 M KOH containing 0.5 M $N_2H_4$ as the anolyte; (b) The correlation of cell voltage with $OH^-$ concentrations in the catholyte at 500 mA cm$^{-2}$.

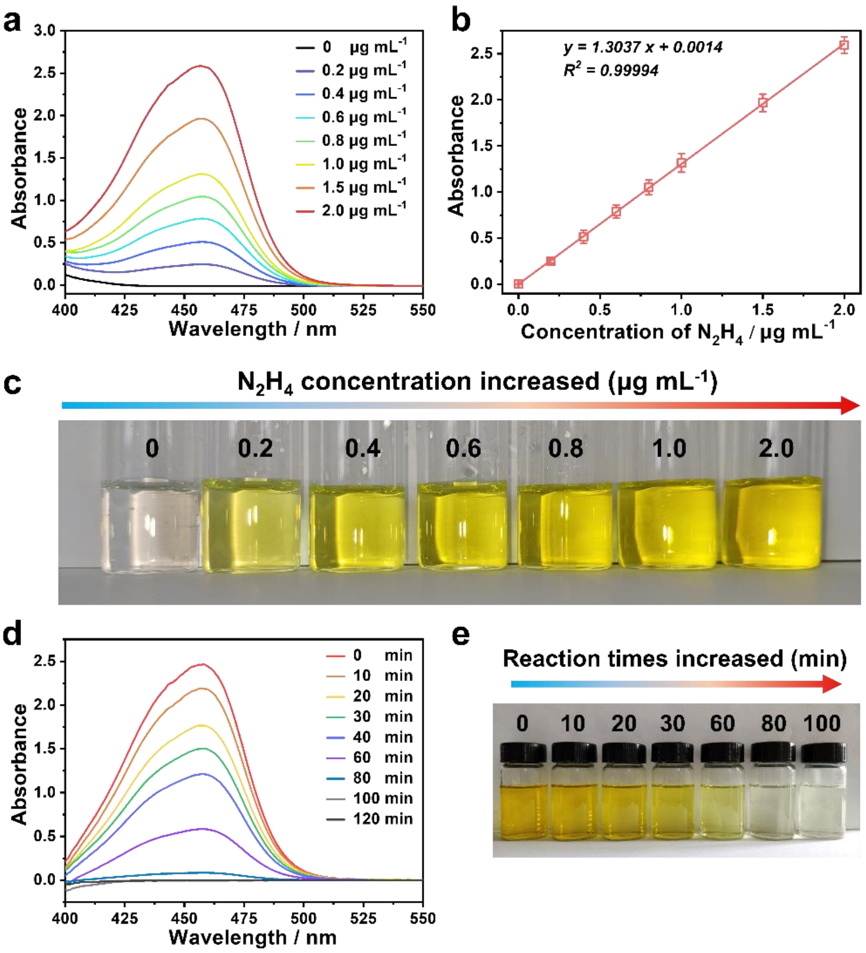


**Supplementary Figure 27.** (a) UV-vis absorption spectra and (b) calibration curve of the colorimetric $N_2H_4$ assay by the Watt and Chrisp method. (c) Optical image of standard $N_2H_4$ solution with various concentrations used for the above tests. (d) UV-vis spectra and (e) optical image of the colorimetric $N_2H_4$ assay of the analyte after electrolysis for different times at 500 mA $cm^{-2}$.

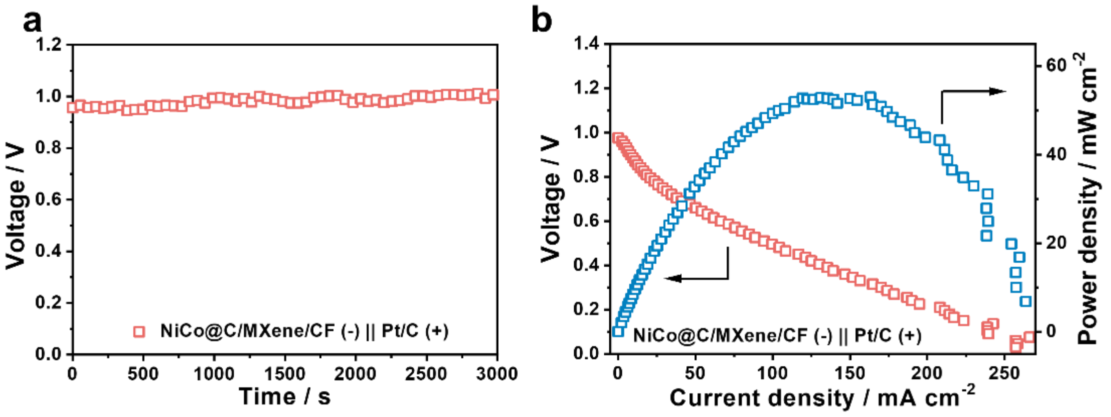


**Supplementary Figure 28.** (a) Open-circuit voltage and (b) discharge polarization and power density curves of this DHzFC.

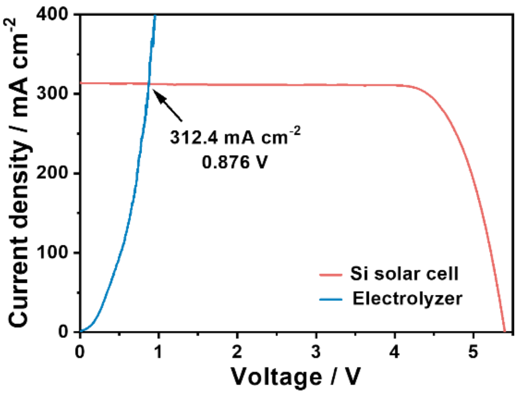


**Supplementary Figure 29.** Current density-voltage curves of HSE and Si-based solar cell connected to it under simulated AM 1.5G (100 mW cm$^{-2}$) illumination.

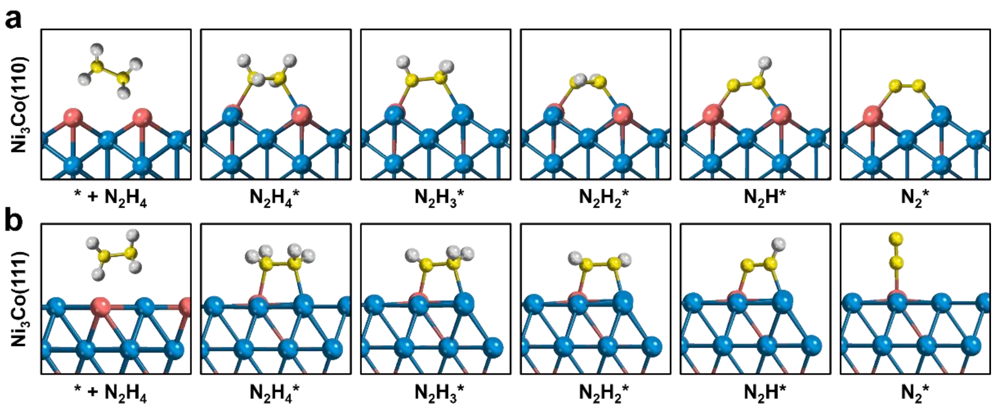


**Supplementary Figure 30.** Structural models of reaction intermediates adsorbed on the (110) and (111) facets of $Ni_3Co$ alloy for stepwise $N_2H_4$ dehydrogenation.

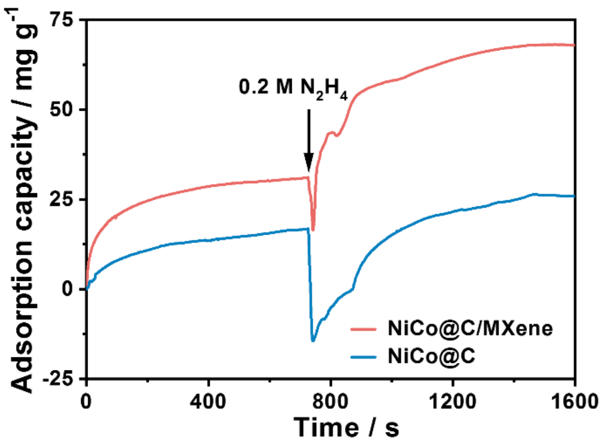


**Supplementary Figure 31.** The mass change of NiCo@C/MXene and NiCo@C during the adsorption of water and hydrazine molecules, which is measured by EQCM. The sudden drop of adsorption capacity upon adding $N_2H_4$ is due to the reduction of oxide species on the electrode surface by hydrazine.

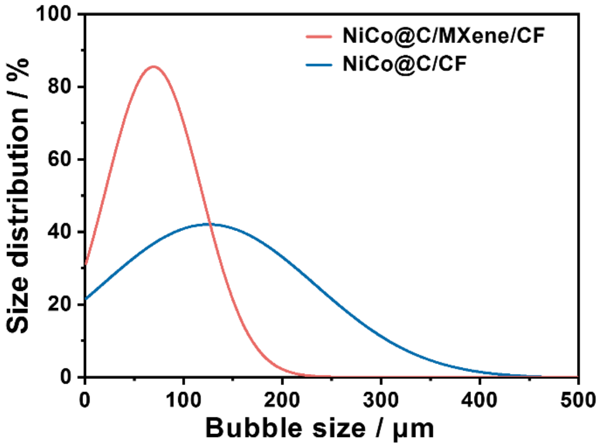


**Supplementary Figure 32.** The size distribution of gas bubbles released from NiCo@C/MXene/CF and NiCo@C/CF during HER in 1.0 M KOH at 20 mA $cm^{-2}$.

**Supplementary Table 1.** A comparison of NiCo@C/MXene/CF with reported 3D electrodes in HzOR performance.

| Catalyst | Electrolyte | Current density (mA cm$^{-2}$) | Potential (mV vs. RHE) | Stability (h) | Reference |
|---|---|---|---|---|---|
| **NiCo@C/MXene/CF** | **1.0 M KOH + 0.5 M $N_2H_4$** | **10**<br>**100**<br>**500** | **-96**<br>**-25**<br>**43** | **30** | **This work** |
| $Ni_2P$/NF | 1.0 M KOH + 0.5 M $N_2H_4$ | 100 | ~ -7 | 10 | *Angew. Chem. Int. Ed. 2017, 56, 842* |
| Ni(Cu) CNPs | 1.0 M KOH + 0.5 M $N_2H_4$ | 100 | 76 | 24 | *J. Mater. Chem. A 2020, 8, 21084* |
| Ni(Cu)@NiFeP/NM | 1.0 M KOH + 0.5 M $N_2H_4$ | 100 | 108 | 12 | *J. Catal. 2019, 373, 180* |
| $CoSe_2$ nanosheets/NF | 1.0 M KOH + 0.5 M $N_2H_4$ | 100 | 170 | – | *Angew. Chem. Int. Ed. 2018, 57, 7649* |
| $Cu_1Ni_2$-N/CC | 1.0 M KOH + 0.5 M $N_2H_4$ | 100 | ~ 205 | 35 | *Adv. Energy Mater. 2019, 9, 1900390* |
| $Ni_3S_2$/NF | 1.0 M KOH + 0.2 M $N_2H_4$ | 100 | 415 | 10 | *J. Mater. Chem. A 2018, 6, 19201* |
| $Ni_3N$-$Co_3N$ PNAs/NF | 1.0 M KOH + 0.1 M $N_2H_4$ | 100 | -9 | 40 | *Angew. Chem. Int. Ed. 2021, 60, 5984* |
| PW-$Co_3N$ NWA/NF | 1.0 M KOH + 0.1 M $N_2H_4$ | 100 | ~ -8 | 10 | *Nat. Commun. 2020, 11, 1853* |

**Supplementary Table 2.** The residue of Ni, Co and Ti ions in the electrolyte after long-term HzOR or HER in alkaline electrolytes.

| **Elements** | **HER**<br>Concentration (mg $L^{-1}$) | **HzOR**<br>Concentration (mg $L^{-1}$) |
|---|---|---|
| Ni | 0.0005 | 0.0002 |
| Co | 0.0007 | 0.0004 |
| Ti | 0.001 | 0.0006 |

**Supplementary Table 3.** A comparison of NiCo@C/MXene/CF with reported 3D electrode in HER performance.

| Catalyst | Electrolyte | Current density (mA $cm^{-2}$) | Overpotential (mV) | Stability (h) | Reference |
|---|---|---|---|---|---|
| **NiCo@C/MXene/CF** | **1.0 M KOH** | **10**<br>**100**<br>**500** | **49**<br>**153**<br>**235** | **60** | **This work** |
| $NiCo_2P_x$/CF | 1.0 M KOH | 10 | 58 | 30 | *Adv. Mater. 2017, 29, 1605502* |
| Ni@NC/NF | 1.0 M KOH | 10 | 61 | 18 | *J. Mater. Chem. A 2019, 7, 8129* |
| $Ni_3N$-VN/NF | 1.0 M KOH | 10 | 59 | 20 | *Adv. Mater. 2019, 31, 1901174* |
| Co@N-CS/N-HCP@CC | 1.0 M KOH | 10 | 66 | 30 | *Adv. Energy Mater. 2019, 9, 1803918* |
| *h*-$Co_{0.34}Fe_{0.33}Ni_{0.33}$-LDH/NF | 1.0 M KOH | 10 | 71 | 25 | *Adv. Mater. 2020, 32, 2006784* |
| $Cu_1Ni_2$-N/CC | 1.0 M KOH | 10 | 71.4 | 60 | *Adv. Energy Mater. 2019, 9, 1900390* |
| $CoSe_2$ nanosheets/NF | 1.0 M KOH | 10 | 79 | 50 | *Angew. Chem. Int. Ed. 2018, 57, 7649* |
| Mo-$Ni_3S_2$/$Ni_xP_y$/NF | 1.0 M KOH | 10 | 109 | 24 | *Adv. Energy Mater. 2020, 10, 1903891* |

**Supplementary Table 4.** Calculation details of energy equivalent input and $CO_2$ equivalent emission for HSE and alkaline water electrolysis.

| | **Alkaline water electrolysis** | | **HSE** | |
|---|---|---|---|---|
| **Content** | **$CO_2$ equivalent emission ($t_{CO2}$ $t_{H2}^{-1}$)** | **Energy equivalent input (GJ $t_{H2}^{-1}$)** | **$CO_2$ equivalent emission ($t_{CO2}$ $t_{H2}^{-1}$)** | **Energy equivalent input (GJ $t_{H2}^{-1}$)** |
| Water extraction & deionization | 0.045 | 0.000117 | 0 | 0 |
| Heating $H_2O$ to 70 °C | 0.01 | 1.696 | 0 | 0 |
| Electrolysis stack | 1.04 | 187.2 | 0.627 | 112.9 |
| Heat demand of reaction | 0.151 | 27.25 | 0 | 0 |
| Overall | 1.246 | 216.146 | 0.627 | 112.9 |

* Note: The data of alkaline water electrolysis and electrochemical splitting methane are obtained from Ref. 15. The data of natural gas steam reforming is obtained from Ref. 16.

**Supplementary Table 4.** A comparison of HSE with reported seawater electrolyzers in cell performance.

| Catalyst | Electrolyte | Current density (mA cm$^{-2}$) | Cell Voltage (V) | Basic electricity expense (kWh m$^{-3}$ $H_2$) | Reference |
|---|---|---|---|---|---|
| **NiCo@C/MXene/CF (+, –)** | **1.0 M KOH + 0.5 M $N_2H_4$ (+) \|\| 1.0 M KOH + seawater (–)** | **500** | **1.15** | **2.75** | **This work** |
| | **1.0 M KOH + 0.5 M $N_2H_4$ (+) \|\| Neutral seawater (–)** | **300** | **1.0** | **2.39** | |
| NiFe-LDH (+) \|\| Pt/C (–) | 0.5 M KOH (+) \|\| 0.5 M NaCl (–) | ~ 275 | 1.7 | 4.06 | *Energy Environ. Sci. 2020,13, 1725* |
| NiMoN@NiFeN/NF (+) \|\| NiMoN/NF (–) | 1.0 M KOH + seawater (+, –) | 500 | 1.8 | 4.3 | *Nat. Commun. 2019, 10, 5106* |
| NiFe/$NiS_x$/Ni (+) \|\| Ni-NiO-$Cr_2O_3$/NF (–) | 1.0 M KOH + seawater (+, –) | 400 | 2.12 | 5.07 | *PNAS 2019, 116, 6624* |
| S-(Ni,Fe)OOH/NF (+) \|\| NiMoN/NF (–) | 1.0 M KOH + seawater (+, –) | 500 | 1.85 | 4.42 | *Energy Environ. Sci. 2020, 13, 3439* |
| NiFe LDH (+) \|\| Pt/C (–) | 1.0 M KOH + 0.5 M NaCl (+, –) | 200 | 1.6 | 3.82 | *Adv. Energy Mater. 2018, 8, 1800338* |

| | | | | | |
|---|---|---|---|---|---|
| MHCM-z-BCC (+) ‖ NiMoS/CC (–) | Buffered seawater (+, –) | 10 | 2.1 | 5.02 | *Adv. Mater. 2018, 30, 1707261* |
| Co-Se1(+) ‖ Co-Se4 (–)* | Seawater (+, –) | 20 | 2.0 | 4.78 | *Adv. Energy Mater. 2018, 8, 1801926* |
| NiNS/NF (+, –)* | Seawater (+, –) | 70 | 2.0 | 4.78 | *J. Mater. Chem. A 2019, 7, 8117* |
| S-(Ni,Fe)OOH/NF(+) ‖ NiCoN\|$Ni_xP$\|NiCoN/NF (–) | Seawater (+, –) | 10 | 1.81 | 4.33 | *ACS Energy Lett. 2020, 5, 2681* |

* Note: the data presented with *iR* correction in the reference. The actual electricity expense should be higher.

**Supplementary Table 5.** The binding energy ($E_b$) of $N_2H_4$ molecule on different facets of $Ni_3Co$ alloy.

| Facet | Conformations | $E_b$ (eV) |
|---|---|---|
| | atop Co | -1.44 |
| $Ni_3Co$ (111) | atop Ni | -1.26 |
| | bidentrate | -1.43 |
| | atop Co | -1.42 |
| $Ni_3Co$ (110) | atop Ni | -1.24 |
| | bidentrate | -1.77 |
| | atop Co | -1.54 |
| $Ni_3Co$ (100) | atop Ni | -1.89 |
| | bidentrate | -1.84 |

**Supplementary References**


1. McCrory, C. C. L., Jung, S., Peters, J. C. & Jaramillo, T. F. Benchmarking heterogeneous electrocatalysts for the oxygen evolution reaction. *J. Am. Chem. Soc.* **135**, 16977-16987 (2013).
2. Li, Y. et al. Partially exposed $RuP_2$ surface in hybrid structure endows its bifunctionality for hydrazine oxidation and hydrogen evolution catalysis. *Sci. Adv.* **6**, eabb4197 (2020).
3. Zhai, P. et al. Engineering active sites on hierarchical transition bimetal oxides/sulfides heterostructure array enabling robust overall water splitting. *Nat. Commun.* **11**, 5462 (2020).
4. Liu, X. et al. Self-powered $H_2$ production with bifunctional hydrazine as sole consumable. *Nat. Commun.* **9**, 4365 (2018).
5. Hsu, S. et al. An earth-abundant catalyst-based seawater photoelectrolysis system with 17.9% solar-to-hydrogen efficiency. *Adv. Mater.* **30**, 1707261 (2018).
6. Kresse, G. & Furthmüller, J. Efficient iterative schemes for ab initio total-energy calculations using a plane-wave basis set. *Phys. Rev. B* **54**, 11169-11186 (1996).
7. Kresse, G. & Joubert, D. From ultrasoft pseudopotentials to the projector augmented-wave method. *Phys. Rev. B* **59**, 1758-1775 (1999).
8. Perdew, J. P., Burke, K. & Ernzerhof, M. Generalized gradient approximation made simple. *Phys. Rev. Lett.* **77**, 3865-3868 (1996).
9. Grimme, S., Antony, J., Ehrlich, S. & Krieg, H. A consistent and accurate ab initio parametrization of density functional dispersion correction (DFT-D) for the 94 elements H-Pu. *J. Chem. Phys.* **132**, 154104 (2010).
10. Feng, G. et al. Atomically ordered non-precious $Co_3Ta$ intermetallic nanoparticles as high-performance catalysts for hydrazine electrooxidation. *Nat. Commun.* **10**, 4514 (2019).
11. Liu, Y. et al. Manipulating dehydrogenation kinetics through dual-doping $Co_3N$ electrode enables highly efficient hydrazine oxidation assisting self-powered $H_2$ production. *Nat. Commun.* **11**, 1853 (2020).
12. Chase, M. W. NIST-JANAF thermochemical tables for oxygen fluorides. *J. Phys. Chem. Ref. Data* **25**, 551-603 (1996).
13. Nørskov, J. K. et al. Origin of the overpotential for oxygen reduction at a fuel-cell cathode. *J. Phys. Chem. B* **108**, 17886-17892 (2004).
14. Wang, Z. et al. Single Ru atoms with precise coordination on a monolayer layered double hydroxide for efficient electrooxidation catalysis. *Chem. Sci.* **10**, 378-384 (2019).

15. Xiao, W. & Fan, Z. Electrochemical splitting of methane in molten salts to produce hydrogen. *Angew. Chem. Int. Ed.* **60**, 7664-7668 (2021).
16. Pamela, L. S. & Margaret K. M. Life cycle assessment of hydrogen production via natural gas steam reforming. U.S. Dep. Energy, NREL/TP-570-27637 (2001). https://www.energy.gov/eere/fuelcells/downloads/life-cycle-assessment-hydrogen-production-natural-gas-steam-reforming
