# Supplementary material for: Energy-saving hydrogen production by chlorine-free hybrid seawater splitting coupling hydrazine degradation
Source: Nat Commun. 2021 Jul 7;12:4182. doi: 10.1038/s41467-021-24529-3 (PMC8263752; doi:10.1038/s41467-021-24529-3)
Supplement: Supplementary file 6 — Description of additional supplementary files [file 41467_2021_24529_MOESM6_ESM.docx]

Description of additional supplementary information files

Title: Supplementary Movie 1.

Description: Self-powered hydrogen production system by integrating a hybrid seawater electrolyzer to direct hydrazine full cell.

Title: Supplementary Movie 2.

Description: Hydrogen production by a hybrid seawater electrolyzer connecting to a solar cell under simulated sunlight.

Title: Supplementary Movie 3.

Description: Hydrogen production by a hybrid seawater electrolyzer connecting to a solar cell under natural light.
